# Polarization-independent circulator based on ferrite and plasma materials in two-dimensional photonic crystal

**DOI:** 10.1038/s41598-018-26189-8

**Published:** 2018-05-18

**Authors:** Xiang Xi, Mi Lin, Wenbiao Qiu, Zhengbiao Ouyang, Qiong Wang, Qiang Liu

**Affiliations:** THz Technical Research Center, Shenzhen Key Laboratory of Micro-Nano Photonic Information Technology, College of Electronic Science and Technology, Shenzhen University, Shenzhen, 518060 China

## Abstract

We propose a type of polarization-independent circulator based on ferrite and plasma materials in a two-dimensional photonic crystal (PhC) slab. First, on the basis of analyzing the wave equations in ferrite and plasma materials, TE and TM circulators are realized with ferrite and plasma in PhCs, respectively. Then, by properly combining these two types of circulators together, a polarization-independent circulator is achieved and investigated. The results show that, for both polarizations, the insertion loss and isolation for the polarization-independent circulator are less than 0.15 dB and more than 20 dB, respectively. Finite-element method is used to calculate the characteristics of the circulators and Nelder-Mead optimization method is employed to obtain the optimized parameters. The idea presented here may have potential applications in integrated photonic circuits and devices.

## Introduction

Circulators are nonreciprocal devices that only allow waves to be transmitted along a specific direction and are widely used in modern communications technology in several critical applications. These devices can be used to protect source signals from the effects of harmful reflections, can extract feedback signals for use in diagnostic or detection systems, and can be used to design add-drop multiplexers or optical band pass filters^1–3^.

Following the development of photonic crystals (PhCs), circulators based on PhCs^4–7^ have been studied actively because of their compactness, suitability for applications, and efficiency. Many high quality circulators have been fabricated based on PhCs. For example, several types of PhC circulators with high isolation and low insertion loss made from bismuth-iron-garnet (BIG) have been proposed^8–10^. In refs^8–10^, the researchers used BIG to construct magneto-optical (MO) cavities and successfully designed three-port, four-port and even six-port circulators for operation at optical frequencies. A type of compact W-format PhC circulator with modulated MO cavities was also proposed^11^ that required only one single-direction external magnetic field and no rotational symmetry. In addition, yttrium-iron-garnet (YIG) has also been used to realize PhC circulators^12–17^. Several circulators with different shapes were fabricated based on the MO effect provided by YIG-based materials, including T-type, Y-type and cross-type circulators. However, all the PhC circulators described above can only operate with a specific polarization type (i.e., TE or TM polarization only), which may greatly restrict their potential applications. In practice, e.g., in biosensing applications, a single-polarization incident wave may produce reflected or transmitted waves that have both TE and TM polarization components. It is therefore necessary to perform further research to produce polarization-independent circulators (PIC) for higher signal processing efficiency.

Up to now, several types of PICs have been proposed. Matsumoto’s group^18^ proposed a kind of optical PIC by using components such as YIG crystals, polarization prisms, compensating plates, and magnet rings. They proved that their circulator obtained 3.7 dB insertion loss and 10–20 dB isolation. Then, Sugimoto *et al*.^19^ proposed a type of waveguide PIC based on the nonreciprocal Mach-Zehnder interferometer. Waveguide rotators, half waveplates and planar 3-dB couplers were used to compose that interferometer. The insertion loss and isolation of their circulator were 3 dB and 20 dB, respectively. After that, a 4-port PIC was fulfilled by the careful assembly of holographic spatial walk-off polarizers^20^, which were built based on the coupled-wave theory. In addition, another waveguide PIC suitable for monolithic integration was proposed^21^ where multimode interferometers, half-wave plates, and rotators in waveguide form were used and 25 dB of isolation and 1.3 dB of insertion loss were obtained in optical regime.

In this paper, a type of PIC that is based on both ferrite and plasma materials^22–30^ in a two-dimensional (2D) triangular-lattice PhC slab is proposed. First, the wave equations in these ferrite and plasma materials are studied. Then, we use the ferrite materials to build a TE PhC circulator, and use the plasma materials to build a TM PhC circulator. Finally, we combine these two circulators together to realize a PIC. The finite-element method (FEM)^31–33^ is then used to calculate the output properties of these structures and the Nelder-Mead optimization method (NOM)^34,35^ is used to optimize the circulator parameters. The greatest advantage of the proposed structure is that it is polarization-independent, which makes it highly efficient in practical applications. For example, in processing mixed-polarization signals in communication systems or circular-polarized signals in biosensors, PIC can decrease the loss or promoting the sensitivity. As another example, in polarization logic systems, PIC is necessary for the operation of both polarizations in the systems. Comparing with the PICs mentioned above, most of the PICs consist of multiple components so that our structure is more compact in size. Besides, the insertion loss of our circulator is much lower. In addition, all the air holes and ferrite or plasma material regions in our PhC slab are cylindrically shaped, which means that the structure is easy to fabricate and feasible in practice.

To the best of our knowledge, it is the first time to realize the PIC in PhC structure with both the ferrite and plasma materials. However, due to the limit of materials at present stage, the circulators studied in this paper are designed for microwave frequencies using experimentally feasible ferrite and plasma materials. Our design can be extended to optical regime when the needed materials become available in the future.

## Theoretical Analysis - Wave Equations in Ferrite and Plasma Materials

### Ferrite Material

First, we consider electromagnetic (EM) waves propagation in the ferrite material. In a source-free region, the wave equations can be deduced based on Maxwell’s differential equations:

1 $$\nabla \times {\bf{H}}=\,j\omega {\varepsilon }_{0}{\varepsilon }_{r}{\bf{E}},$$

2 $$\nabla \times {\bf{E}}=-\,j\omega {\mu }_{0}{\mu }_{r}{\bf{H}}.$$

By considering the vector identity $\nabla \times \nabla \times {\bf{H}}=\nabla (\nabla \cdot {\bf{H}})-{\nabla }^{2}{\bf{H}}$, the wave equation for ${\bf{H}}$ can be obtained from Eqs (1) and (2),

3 $${\nabla }^{2}{\bf{H}}-\nabla (\nabla \cdot {\bf{H}})+{\omega }^{2}{\varepsilon }_{0}{\varepsilon }_{r}{\mu }_{0}{\mu }_{r}\cdot {\bf{H}}=0.$$

Under an external direct-current (DC) magnetic field, the relative permittivity of the ferrite material ${\varepsilon }_{r}$ is known to be constant, while its relative permeability has the following tensor form

4 $$[{\mu }_{r}]=[\begin{array}{ccc}{\mu }_{11} & {\mu }_{12} & {\mu }_{13}\\ {\mu }_{21} & {\mu }_{22} & {\mu }_{23}\\ {\mu }_{31} & {\mu }_{32} & {\mu }_{33}\end{array}].$$

It is assumed that the electric field and the magnetic field in the ferrite material take the forms of ${\bf{E}}=({E}_{x},{E}_{y},{E}_{z}){e}^{-j{\bf{k}}\cdot {\bf{r}}}$ and ${\bf{H}}=({H}_{x},{H}_{y},{H}_{z}){e}^{-j{\bf{k}}\cdot {\bf{r}}}$, respectively, where ${\bf{k}}={k}_{x}{{\bf{e}}}_{x}+{k}_{y}{{\bf{e}}}_{y}+{k}_{z}{{\bf{e}}}_{z}$ is the wave vector, ${\bf{r}}=x{{\bf{e}}}_{x}+y{{\bf{e}}}_{y}+z{{\bf{e}}}_{z}$ is the position vector, and $({{\bf{e}}}_{x},{{\bf{e}}}_{y},{{\bf{e}}}_{z})$ is the unit vector. The following matrix form of Eq. (3) can then be derived:

5 $${k}^{2}[\begin{array}{c}{H}_{x}\\ {H}_{y}\\ {H}_{z}\end{array}]-[\begin{array}{ccc}{{k}_{x}}^{2} & {k}_{x}{k}_{y} & {k}_{x}{k}_{z}\\ {k}_{y}{k}_{x} & {{k}_{y}}^{2} & {k}_{y}{k}_{z}\\ {k}_{z}{k}_{x} & {k}_{z}{k}_{y} & {{k}_{z}}^{2}\end{array}]\,[\begin{array}{c}{H}_{x}\\ {H}_{y}\\ {H}_{z}\end{array}]-{{k}_{0}}^{2}{\varepsilon }_{r}[\begin{array}{ccc}{\mu }_{11} & {\mu }_{12} & {\mu }_{13}\\ {\mu }_{21} & {\mu }_{22} & {\mu }_{23}\\ {\mu }_{31} & {\mu }_{32} & {\mu }_{33}\end{array}]\,[\begin{array}{c}{H}_{x}\\ {H}_{y}\\ {H}_{z}\end{array}]=0.$$

where $k=\sqrt{{k}_{x}^{2}+{k}_{y}^{2}+{k}_{z}^{2}}$ is the propagation constant of the ferrite material and ${k}_{0}=\omega \sqrt{{\varepsilon }_{0}{\mu }_{0}}$ is the propagation constant of air.

**Figure 1:**
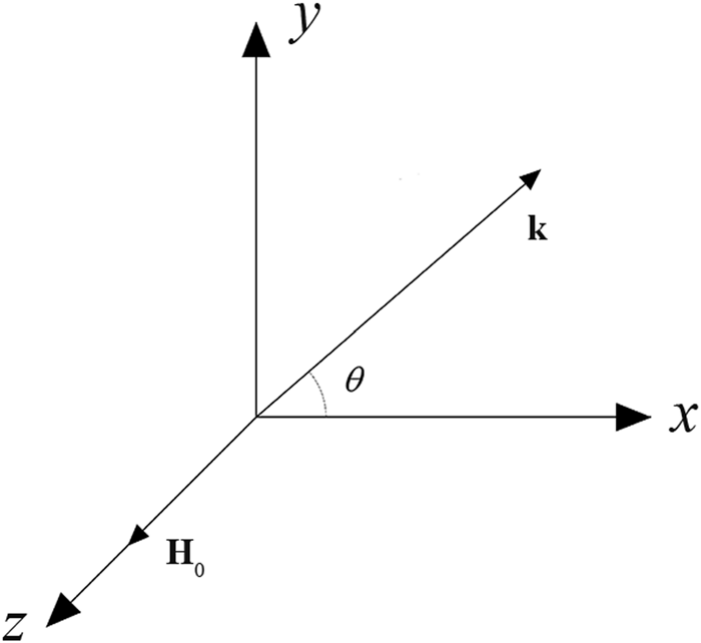
The schematic diagram of the coordinate system, where the directions of the wave vector **k** and the external magnetic field **H**_0_ are indicated.

Equation (5) describes the properties of the magnetic field **H** in the ferrite material. In the following, we solve this equation under several different conditions to enable discussion of the behaviour of the EM wave. For simplicity, we assume that the wave propagates along the *x-y* plane at an angle *θ* and that the external DC magnetic field is applied along the *z*-direction as shown in Fig. 1.

#### TE waves propagation (non-magnetized ferrite)

When a TE wave propagates along the *x-y* plane at an angle *θ*, the electric field is oriented parallel to the *z*-axis while the magnetic field is along the *x-y* plane. We therefore have ${\bf{E}}=(0,0,{E}_{z}){e}^{-j{\bf{k}}\cdot {\bf{r}}}$, ${\bf{H}}=({H}_{x},{H}_{y},0){e}^{-j{\bf{k}}\cdot {\bf{r}}}$, and ${\bf{k}}={k}_{x}{{\bf{e}}}_{x}+{k}_{y}{{\bf{e}}}_{y}$, where *k*_*x*_ **=** *k* cos*θ* and *k*_*y*_ **=** *k* sin*θ*.

When no external DC magnetic field is applied, the ferrite material is un-magnetized and its relative permeability can be simplified to the following form^36^

6 $$[{\mu }_{r}]=[\begin{array}{ccc}{\mu }_{C} & 0 & 0\\ 0 & {\mu }_{C} & 0\\ 0 & 0 & {\mu }_{C}\end{array}].$$

where *μ*_*C*_ is a constant that is dependent on the type of ferrite materials used.

Under these conditions, Eq. (5) can be written in a simplified form as:

7 $$[\begin{array}{cc}{k}^{2}{\sin }^{2}\theta -{k}_{0}^{2}{\varepsilon }_{r}{\mu }_{C} & -{k}^{2}\,\cos \,\theta \,\sin \,\theta \\ -{k}^{2}\,\cos \,\theta \,\sin \,\theta  & {k}^{2}{\cos }^{2}\theta -{k}_{0}^{2}{\varepsilon }_{r}{\mu }_{C}\end{array}]\,[\begin{array}{c}{H}_{x}\\ {H}_{y}\end{array}]=0.$$

By solving Eq. (7) and using the differential equation that relates ${\bf{H}}$ to ${\bf{E}}$ in Eq. (1), we obtain the following relationships:

8 $${k}^{2}={k}_{0}^{2}{\varepsilon }_{r}{\mu }_{C},$$

9 $$\frac{{H}_{x}}{{H}_{y}}=\frac{{k}^{2}\,\cos \,\theta \,\sin \,\theta }{{k}^{2}{\sin }^{2}\theta -{k}_{0}^{2}{\varepsilon }_{r}{\mu }_{C}},$$

10 $${E}_{x}={E}_{y}={H}_{z}=0,$$

11 $${E}_{z}=\sqrt{\frac{{\mu }_{0}{\mu }_{C}}{{\varepsilon }_{0}{\varepsilon }_{r}}}({H}_{x}\,\sin \,\theta \,-\,{H}_{y}\,\cos \,\theta ).$$

Traditionally, the polarization is regarded as the direction of the electric vectors and it is thus also called the electric polarization. However, to outline the behaviour of the wave more clearly here, we also use the magnetic polarization, i.e., the direction of the magnetic vectors. From Eq. (8), we obtain $k={k}_{0}\sqrt{{\varepsilon }_{r}{\mu }_{C}}=\omega \sqrt{{\varepsilon }_{0}{\mu }_{0}{\varepsilon }_{r}{\mu }_{C}}=n\omega /c$, where *c* is the speed of light in a vacuum and $n=\sqrt{{\varepsilon }_{r}{\mu }_{C}}$ is the effective refractive index of the ferrite materials. On the one hand, from Eqs (9–11), we find that there is only the ${E}_{z}$ component for the electric field and both ${H}_{x}$ and ${H}_{y}$ components for the magnetic field, which means that the TE wave can propagate in the un-magnetized ferrite material; on the other hand, ${H}_{x}/{H}_{y}$ obviously has a real form, which means that there is no phase difference between ${H}_{x}$ and ${H}_{y}$. Therefore, the magnetic field ${\bf{H}}$ is linearly polarized. As a result, the wave propagation direction will remain unchanged. The un-magnetized ferrite material acts as an isotropic material and will not provide a rotation effect for the TE wave.

#### TE waves propagation (magnetized ferrite)

Similarly, in this case we start with $${\bf{E}}=(0,0,{E}_{z}){e}^{-j{\bf{k}}\cdot {\bf{r}}}$$, $${\bf{H}}=({H}_{x},$$$${H}_{y},0){e}^{-j{\bf{k}}\cdot {\bf{r}}}$$, and $${\bf{k}}={k}_{x}{{\bf{e}}}_{x}+{k}_{y}{{\bf{e}}}_{y}$$. When an external DC magnetic field is applied in the *z*-direction, the ferrite material becomes magnetized and its relative permeability can then be expressed as^36^

12 $$[{\mu }_{r}]=[\begin{array}{ccc}{\mu }_{m} & \pm j{\mu }_{k} & 0\\ \mp j{\mu }_{k} & {\mu }_{m} & 0\\ 0 & 0 & {\mu }_{C}\end{array}].$$

where ${\mu }_{m}$ and ${\mu }_{k}$ are frequency-dependent and affected by the intensity of the external magnetic field (their detail expressions are given in the next section). The signs $\pm$ and $\mp$ indicate the external fields in the +*z* and −*z* directions, respectively.

Using these conditions, Eq. (5) can then be reduced as follows:

13 $$[\begin{array}{cc}{k}^{2}{\sin }^{2}\theta -{k}_{0}^{2}{\varepsilon }_{r}{\mu }_{m} & -{k}^{2}\,\sin \,\theta \,\cos \,\theta \mp j{k}_{0}^{2}{\varepsilon }_{r}{\mu }_{k}\\ -{k}^{2}\,\sin \,\theta \,\cos \,\theta \pm j{k}_{0}^{2}{\varepsilon }_{r}{\mu }_{k} & {k}^{2}\,\cos \,{}^{2}\theta -{k}_{0}^{2}{\varepsilon }_{r}{\mu }_{m}\end{array}]\,[\begin{array}{c}{H}_{x}\\ {H}_{y}\end{array}]=0.$$

From Eq. (13) and the differential equation of Eq. (1), we then have

14 $${\mu }_{m}{k}^{2}={k}_{0}^{2}{\varepsilon }_{r}({\mu }_{m}^{2}-{\mu }_{k}^{2}),$$

15 $$\frac{{H}_{x}}{{H}_{y}}=\frac{{k}^{2}\,\sin \,\,\theta \,\cos \,\theta }{{k}^{2}{\sin }^{2}\theta -{k}_{0}^{2}{\varepsilon }_{r}{\mu }_{m}}\pm j\frac{{k}_{0}^{2}{\varepsilon }_{r}{\mu }_{k}}{{k}^{2}{\sin }^{2}\theta -{k}_{0}^{2}{\varepsilon }_{r}{\mu }_{m}},$$

16 $${E}_{x}={E}_{y}={H}_{z}=0,$$

17 $${E}_{z}=\sqrt{\frac{{\mu }_{0}}{{\varepsilon }_{0}{\varepsilon }_{r}}\frac{{{\mu }_{m}}^{2}-{{\mu }_{k}}^{2}}{{\mu }_{m}}}({H}_{x}\,\sin \,\theta -{H}_{y}\,\cos \,\theta ).$$

Equation (14) is the mode-equation of the TE wave in the magnetized ferrite material. The mode equation is a function of frequency that is dependent on the parameters of the ferrite material and takes an identical form for the external magnetic fields in both the +*z* and −*z* directions. From Eqs (15–17), we find that the electric field has only one component, *E*_*z*_, while the magnetic field has both *H*_*x*_ and *H*_*y*_ components, so the TE wave can also propagate in the magnetized ferrite material. However, unlike the magnetic field in the un-magnetized scenario, Eq. (15) also shows that *H*_*x*_/*H*_*y*_ takes complex forms so that *H*_*x*_ and *H*_*y*_ have different phases, i.e., now the magnetic field **H** is elliptically polarized. Therefore, the wave propagation direction will change by noting that the direction of electric field will fix at the *z*-direction but the direction of magnetic field will change along the elliptical path within the *x-y* plane. The magnetized ferrite material provides the rotation effect for the TE wave.

On the other hand, we note that the imaginary part of *H*_*x*_/*H*_*y*_ is negative or positive for external fields in the +*z* and −*z* directions, respectively, and as a result, the phase of *H*_*x*_ is behind or ahead of that of *H*_*y*_. Therefore, the resultant magnetic fields **H** are left-hand or right-hand elliptically polarized around the *z*-axis, respectively. Therefore, according to the right-hand law, the wave propagation directions will change along the clockwise or anti-clockwise directions, respectively, in these two situations. That is the reason why we can always change the circulation direction of a circulator by simply changing the direction of the external magnetic field.

#### TM waves propagation (non-magnetized ferrite)

For a TM wave propagating along the *x-y* plane at an angle *θ*, the magnetic field is oriented parallel to the *z*-axis and the electric field is along the *x-y* plane, i.e., we have ${\bf{E}}=({E}_{x},{E}_{y},0){e}^{-j{\bf{k}}\cdot {\bf{r}}}$, ${\bf{H}}=(0,0,{H}_{z}){e}^{-j{\bf{k}}\cdot {\bf{r}}}$ and ${\bf{k}}={k}_{x}{{\bf{e}}}_{x}+{k}_{y}{{\bf{e}}}_{y}$. The relative permeability of the un-magnetized ferrite material is the same as that used in Eq. (6). Using these conditions, we solve Eq. (5) and obtain the following expressions:

18 $$(1-\frac{{k}^{2}}{{k}_{0}^{2}{\varepsilon }_{r}{\mu }_{C}}){H}_{z}=0,$$

19 $${H}_{x}={H}_{y}={E}_{z}=0,$$

20 $${E}_{x}=-\,\sqrt{\frac{{\mu }_{0}{\mu }_{C}}{{\varepsilon }_{0}{\varepsilon }_{r}}}{H}_{z}\,\sin \,\theta ,$$

21 $${E}_{y}=\sqrt{\frac{{\mu }_{0}{\mu }_{C}}{{\varepsilon }_{0}{\varepsilon }_{r}}}{H}_{z}\,\cos \,\theta .$$

From the above expressions, it is obvious that $1-{k}^{2}/{k}_{0}^{2}{\varepsilon }_{r}{\mu }_{C}=0$ and *H*_*z*_ **≠** 0 otherwise **H** = **E** = 0, which is a trivial or useless solution of the above equations. The magnetic field **H** has only one component, *H*_*z*_, while the electric field **E** has components *E*_*x*_ and *E*_*y*_, so the TM wave can propagate in the un-magnetized ferrite material. In addition, it can easily be found that *E*_*x*_/*E*_*y*_ has a real form, which means that the electric field is linearly polarized. Therefore, the wave propagation direction will remain unchanged. The un-magnetized ferrite material cannot provide a rotation effect for the TM wave in this case.

#### TM waves propagation (magnetized ferrite)

In this case, we begin with ${\bf{E}}=({E}_{x},{E}_{y},0){e}^{-j{\bf{k}}\cdot {\bf{r}}}$, ${\bf{H}}=(0,0,{H}_{z}){e}^{-j{\bf{k}}\cdot {\bf{r}}}$ and ${\bf{k}}={k}_{x}{{\bf{e}}}_{x}+{k}_{y}{{\bf{e}}}_{y}$. Based on the relative permeability of the magnetized ferrite material from Eq. (12), we obtain the following relationships based on Eqs (1) and (5):

22 $$(1-\frac{{k}^{2}}{{k}_{0}^{2}{\varepsilon }_{r}{\mu }_{C}}){H}_{z}=0,$$

23 $${H}_{x}={H}_{y}={E}_{z}=0,$$

24 $${E}_{x}=-\sqrt{\frac{{\mu }_{0}{\mu }_{C}}{{\varepsilon }_{0}{\varepsilon }_{r}}}{H}_{z}\,\sin \,\theta ,$$

25 $${E}_{y}=\sqrt{\frac{{\mu }_{0}{\mu }_{C}}{{\varepsilon }_{0}{\varepsilon }_{r}}}{H}_{z}\,\cos \,\theta .$$

From these equations, we find that all components of ${\bf{H}}$ and ${\bf{E}}$ in the magnetized ferrite material are the same as the corresponding components in the un-magnetized material case. A TM wave can propagate in the magnetized ferrite material but the direction of this wave will remain unchanged. These results also indicate that when the wave propagation direction is perpendicular to the external DC magnetic field, neither the magnetized ferrite nor the un-magnetized ferrite material will provide a rotation effect for the TM wave.

Overall, we found that the un-magnetized ferrite material acted as an isotropic material and it could not provide a rotation effect for both polarizations. The magnetized ferrite material, however, could only provide a rotation effect for the TE wave, and not for the TM wave. Therefore, we will use the ferrite material under a specific external magnetic field to fabricate the TE PhC circulator.

### Plasma Material

We noted that the ferrite material, which has a tensor form for its relative permeability, can provide a rotation effect for TE waves. Based on the symmetry of Maxwell’s wave equations in electric and magnetic vectors^37^, it is a reasonable deduction that the plasma material, which has a tensor form for its relative permittivity, can provide a rotation effect for TM waves. The idea can also be obtained through analysis of the wave equations in the plasma material^28,30^. We omit the detailed steps of this analysis here because they can be easily obtained by a similar way as that in ferrite material. In the following, we will use the plasma material to build our TM PhC circulator.

It should be pointed out that the rotation effect mentioned in this paper refers to the Voigt effect^38^. We only discuss the Voigt effect in MO materials in which the wave propagation direction is perpendicular to the applied DC magnetic field, different from that in Faraday Effect^39^ where the propagation direction is parallel to the applied DC magnetic field.

## Results and Discussions

In the following, we describe the fabrication of the TE, TM, and polarization-independent PhC circulators. While TE and TM PhC circulators have been studied in the published literature^8–17^, we still show these two circulator types here for two reasons. The first is for purposes of comparison. We can compare the performance of the PIC with that of the TE and TM circulators to provide a better understanding of the mechanisms behind these circulators. The second reason is because this approach allows us to present our complete set of design steps for the final PIC.

### TE PhC Circulator

Based on the analyses above, we first use ferrite materials to build a PhC circulator for TE polarization operation.

**Figure 2:**
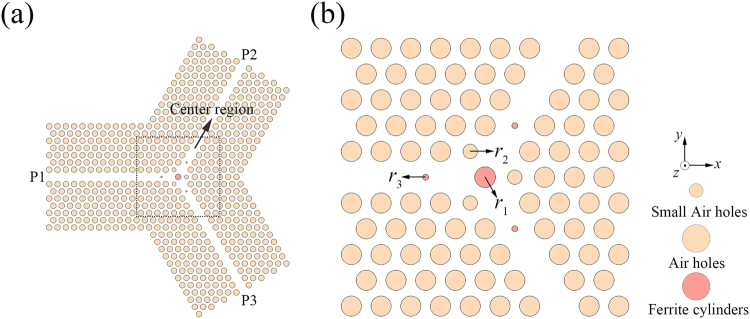
(**a**) Schematic of the TE PhC circulator, and (**b**) magnified view of the centre region.

Schematic of the TE PhC circulator is shown in Fig. 2. The 2D triangular-lattice PhC structure is formed by drilling holes in a silicon slab. The pink and red cylinders represent the air holes and the ferrite cylinders, respectively. In this case, a large ferrite cylinder (denoted by *r*_1_) is introduced into the centre region to provide the rotation effect and the air holes around the central ferrite cylinder are also modulated to a small size (denoted by *r*_2_) to adjust the coupling conditions of the centre micro-cavity. Three additional small ferrite cylinders are also inserted into the waveguides (denoted by *r*_3_) for two reasons. The first reason is that they provide an additional rotation effect; the second reason is that they can be used to adjust the coupling conditions between the centre micro-cavity and the waveguides. It should be noted here that we can also use the other shapes of ferrite material rather than the ferrite cylinders shown in the waveguides to attain our goals; however, the cylinder is the simplest one and much easier to fabricate.

YIG is chosen as ferrite material here, and is the same as that used in refs^12–17^. The relative permittivity of YIG is given as *ε*_r_ = 12.9. In the microwave range, the relative permeability of YIG takes the form of a second-rank tensor under an external DC magnetic field applied in the +*z*-direction^36^:

26 $$[{\mu }_{r}]=[\begin{array}{ccc}{\mu }_{m} & j{\mu }_{k} & 0\\ -j{\mu }_{k} & {\mu }_{m} & 0\\ 0 & 0 & {\mu }_{C}\end{array}].$$

where $${\mu }_{m}=1+{\omega }_{m}({\omega }_{0}+i\alpha \omega )/[{({\omega }_{0}+i\alpha \omega )}^{2}-{\omega }^{2}]$$, $${\mu }_{k}={\omega }_{m}\omega /[{({\omega }_{0}+i\alpha \omega )}^{2}-{\omega }^{2}]$$ and $${\mu }_{C}=1$$ with $${\omega }_{0}=$$$${\mu }_{0}\gamma {H}_{0}$$, $${\omega }_{m}={\mu }_{0}\gamma {M}_{s}$$, and $$\gamma =1.759\times {10}^{11}$$ C/kg. Saturation magnetization of $${M}_{s}=2.39\times {10}^{5}$$ A/m and a loss coefficient of $$\alpha =3\times {10}^{-5}$$ are considered. The applied magnetic field is set here to be $${H}_{0}=3.7\times {10}^{5}$$ A/m.

**Figure 3:**
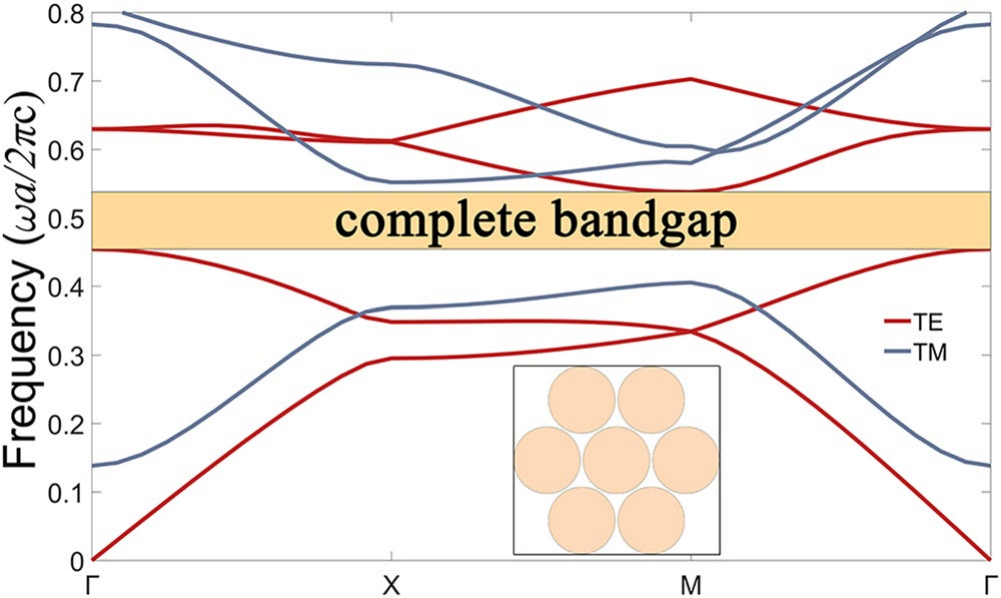
The band structure of the triangle-lattice PhC with air holes embedded in silicon slab for *r*_0_ = 0.48*a*, where the yellow region is the complete bandgap from 0.454 to 0.538 (*ωa*/2πc).

In this paper, the refractive index of the silicon slab is set at *n*_0_ = 3.4. It is well known that there is no complete bandgap if the silicon rods are arranged in an air background to form the triangle-lattice PhC structure. However, a triangular-lattice PhC structure composed of air holes embedded in a silicon slab can have a complete bandgap because the high-*ε* regions of this structure are isolated to a considerable degree, and the triangle-lattice structure has a more circle like Brillouin zone in which the gaps are more likely to open across all the symmetry points^7,40–42^. Here, we select the radius of the air holes *r*_0_ = 0.48*a* with a lattice constant of *a* = 10 mm to obtain the largest complete bandgap. As shown in Fig. 3, the normalized frequency range of complete bandgap is from 0.454 to 0.538 (*ωa*/2πc), corresponding to 1.362 × 10^10^~1.614 × 10^10^ Hz in the microwave band. In the following, we will focus on this frequency range.

**Figure 4:**
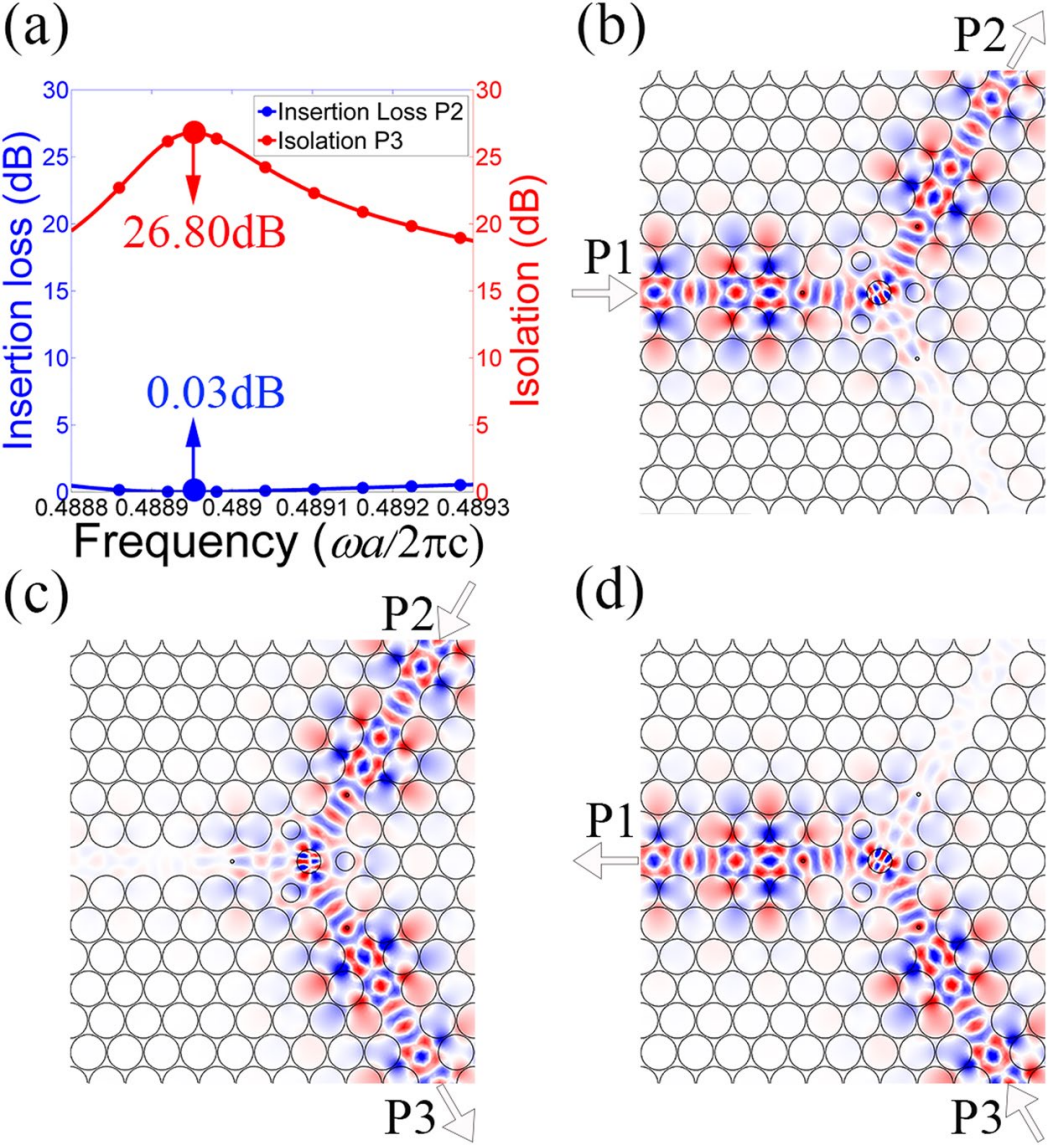
(**a**) The insertion loss and isolation for the TE PhC circulator, and distributions of electric field *E*_z_ in the centre region for (**b**) input from P1, (**c**) input from P2, and (**d**) input from P3.

Obviously, the structural parameters of the centre ferrite cylinder, the small ferrite cylinders in the waveguides, and the air holes around the central ferrite cylinder are crucial to the properties of our TE PhC circulator. Therefore, we use the NOM optimization method to optimize these parameters. The radii of them are obtained to be *r*_1_ = 0.3229*a*, *r*_2_ = 0.0480*a*, and *r*_3_ = 0.2533*a*, respectively, while the distance from the small ferrite cylinder to the centre point is 2.0861*a*, and the distance from the modulated air hole to the centre point is 0.9907*a*. Using these parameters, the resulting frequency response and field distributions are shown in Fig. 4. It should be noted here that we plot the field distributions only in the centre region in order to show the details.

Because of the rotational symmetry of the structure, any arbitrary port can be selected as the input port. Here we select P1 as the input port, and P2 is then the output port while P3 is the isolated port. The insertion loss and the isolation can be calculated using the expressions ‘Insertion Loss (dB) = 10 log_10_ (1/*P*_out_)’ and ‘Isolation (dB) = 10 log_10_ (1/*P*_iso_)’, where *P*_out_, and *P*_iso_ are powers that are normalized with respect to the input power measured at the output and isolated port, respectively. Figure 4(a) shows the insertion loss and isolation for the TE PhC circulator. The best insertion loss is obtained as low as 0.03 dB and the isolation is 26.80 dB at a normalized frequency of 0.48895 (*ωa*/2πc), corresponding to 1.467 × 10^10^ Hz. The field distributions at this frequency are also plotted, as shown in Fig. 4(b–d). The TE PhC circulator works quite well as expected. The waves launched from the input port are transmitted to the output port, almost without any power to the isolated ports. The field distributions prove that the ferrite material provides a rotation effect for the TE polarization and these results fit well with our analysis of the wave equations in the ferrite material.

### The TM PhC Circulator

After obtaining the TE PhC circulator, we then use the plasma materials to build a PhC circulator for the TM polarization.

**Figure 5:**
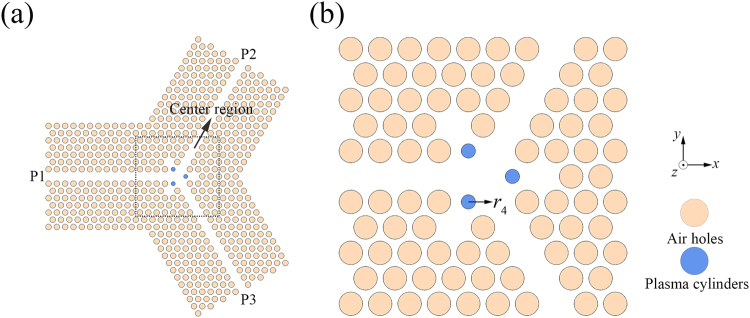
(**a**) Schematic of the TM PhC circulator, and (**b**) magnified view of the centre region.

The schematic of the TM PhC circulator is shown in Fig. 5. The 2D PhC structure is also formed using an array of air holes in a silicon slab. The pink and blue cylinders represent the air holes and the plasma cylinders (denoted by *r*_4_), respectively. Here, the air holes that are located behind the plasma cylinders are removed to form three resonant cavities. In this way, we can promote interaction between the waves and the plasma materials and thus enhance the rotation effect to improve the circulator’s performance. It should be noted here that we could also improve the coupling conditions between the central micro-cavity and the waveguides through the similar way with that in TE PhC circulator by inserting additional plasma cylinders into the waveguides. However, because our ultimate purpose here is to build a PIC and the ferrite cylinders have been inserted into the waveguides, we will not insert any additional plasma cylinders into the waveguides in the TM PhC circulator.

So called plasma material is the full name of plasma, which is a kind of dispersive medium consisting of positive (and negative) ions and elections as well as neutral species. The plasma can be obtained by parallel-plate dielectric barrier excitation, multicapillary electrode discharging, cold cathode fluorescent lamp discharging, gas discharging, and so on. Also, heavily doped semiconductor materials can be regarded as plasma materials^25,43–46^. It is noted that plasma materials have been used for bandgap devices^22,25^, PhC waveguides^44^ and PhC filters^45^ in theory and experiment where characteristic parameters of some plasma materials can be found. When an external magnetic field is applied, the magnetized plasma becomes anisotropic, dispersive, and dissipative, which depends mainly on the external magnetic fields. In this paper, the relative permeability of the plasma material is 1. Under an external DC magnetic field applied in the + *z*-direction, its relative permittivity can be expressed as^46^:

27 $$[{\varepsilon }_{r}]=[\begin{array}{ccc}{\varepsilon }_{m} & j{\varepsilon }_{k} & 0\\ -j{\varepsilon }_{k} & {\varepsilon }_{m} & 0\\ 0 & 0 & {\varepsilon }_{p}\end{array}].$$

where $${\varepsilon }_{m}=1-{{\omega }_{p}}^{2}(\omega -jv)/\omega ({(\omega -jv)}^{2}-{{\omega }_{c}}^{2})$$, $${\varepsilon }_{k}=-\,{\omega }_{c}{{\omega }_{p}}^{2}/\omega ({(\omega -jv)}^{2}-{{\omega }_{c}}^{2})$$ and $${\varepsilon }_{p}=1-{{\omega }_{p}}^{2}/\omega $$$$(\omega -jv)$$. In these expressions, $${\omega }_{p}={({e}^{2}{n}_{e}/{\varepsilon }_{0}m)}^{1/2}$$ is the plasma frequency, where *e*, *m*, *n*_e_, and *ε*_0_ represent electron charge, electron mass, plasma density, and dielectric constant in vacuum, respectively; $${\omega }_{c}=(eB/m)$$ is the cyclotron frequency of electron; and *v* is the plasma frequency. The parameters in [*ε*_*r*_] are decided by *H*_0_, *n*_e_, and *v*. *H*_0_ can be taken according to practical condition. From refs^25–29,44,45^, *n*_e_ can be taken as 1 × 10^11^ ~ 5 × 10^13^ cm^−3^ and *v* = 1 × 10^6^ ~ 1 × 10^10^ Hz. Here we take the external magnetic field as *H*_0_ = 3.7 × 10^5^ A/m, *n*_e_ = 10^13^ cm^−3^ and *v* = 1 × 10^−5^*ω*_p_, which are carefully chosen according to the above mentioned literatures.

**Figure 6:**
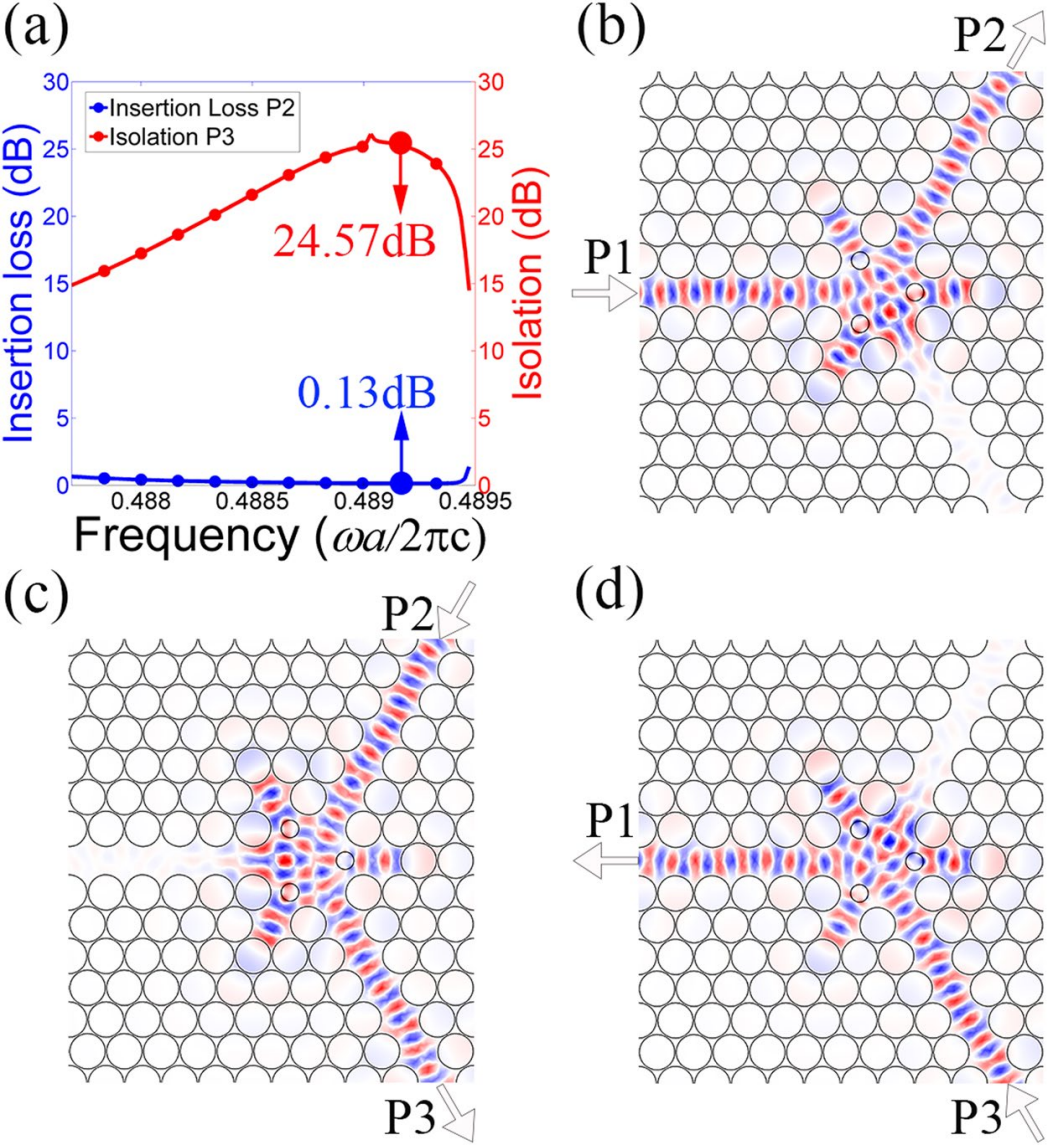
(**a**) The insertion loss and isolation for the TM PhC circulator, and distributions of magnetic field *H*_z_ in the centre region for (**b**) input from P1, (**c**) input from P2, and (**d**) input from P3.

Under these conditions, the frequency responses and the field distributions of the TM PhC circulator are obtained as shown in Fig. 6, in which all field distributions are only plotted in the centre region to show the details more clearly. Here, the radius of the plasma cylinders is *r*_4_ = 0.2395*a*, while the distance from the plasma cylinder to the centre point is 1.001*a*. Figure 6(a) shows that an insertion loss as low as 0.13 dB can be achieved and the isolation is 24.57 dB at a normalized frequency of 0.4892 (*ωa*/2πc), corresponding to 1.468 × 10^10^ Hz. The field distributions at this frequency indicate that this structure realizes near complete transmission from the input port to the output port and almost zero transmission to the isolated port, which shows that the plasma material fulfils its role very well and that the TM PhC circulator performs as expected. This fact demonstrates our analysis that the plasma material provides the rotation effect for the TM polarization.

### Combined Structure: Polarization-Independent Circulator

In this section, we will combine the TE and TM circulators to realize a PIC using both the ferrite and plasma materials.

**Figure 7:**
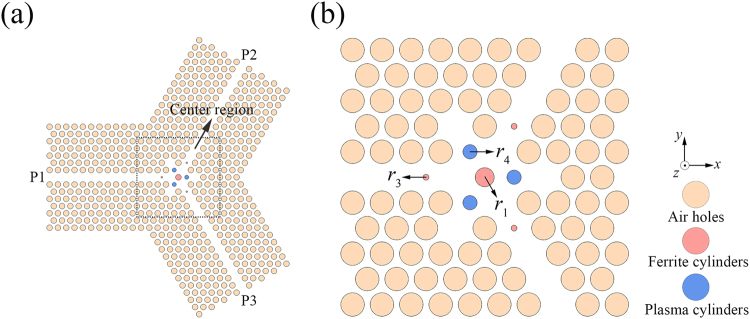
(**a**) Schematic of the PIC, and (**b**) magnified view of the centre region.

The schematic of the PIC is shown in Fig. 7, in which the pink, red, and blue cylinders represent the air holes, the ferrite cylinders, and the plasma cylinders, respectively. The material parameters of the ferrite and plasma materials are chosen as per the previous sections. Because of the changes in the structure, the structural parameters have to be adjusted slightly. We also use the NOM optimization method to obtain the parameters here, and the optimized parameters are *r*_1_ = 0.3210*a*, *r*_3_ = 0.0365*a*, and *r*_4_ = 0.2428*a*; the distance from the small ferrite cylinder to the centre point is 2.0946*a*; and the distance from the plasma cylinder to the centre point is 0.9980*a*.

**Figure 8:**
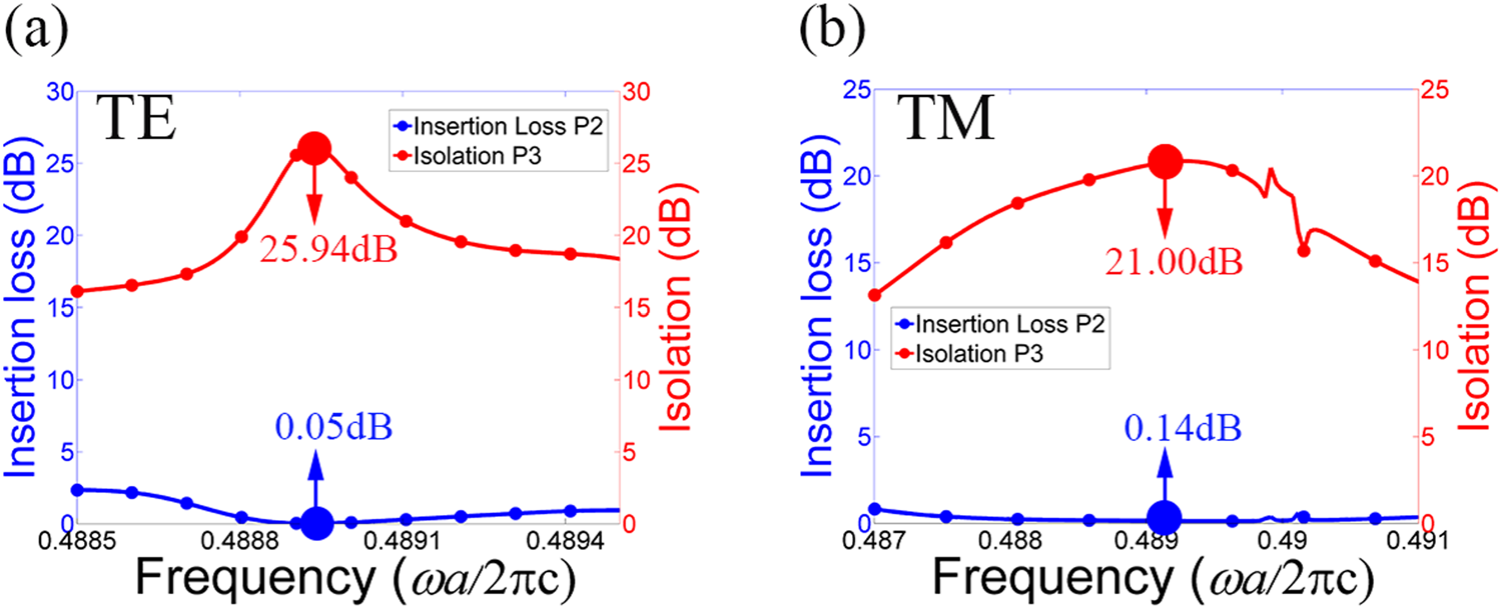
The insertion loss and isolation of the PIC for (**a**) the TE polarization, and (**b**) the TM polarization.

The insertion loss and the isolation are calculated as shown in Fig. 8. For the TE polarization, the lowest insertion loss obtained is 0.05 dB and the isolation reaches a highest value of 25.94 dB; for TM polarization, the optimized insertion loss and the corresponding isolation are 0.14 dB and 21.0 dB, respectively. It should be note here that the performance of this circulator is slightly worse than that in the TE or TM PhC circulator because we should balance the performance for both polarizations in PIC.

We note from Fig. 8 that the insertion loss and isolation for both TE and TM modes have frequency dependence. Such frequency dependence is an integrated result of the dispersions of ferrite, plasma, the cavity and waveguides in the system.

If we define the insertion loss less than 0.5 dB and the isolations greater than 15 dB as the operation condition, the normalized operation bandwidth of our circulator is from 0.4887 to 0.4892 (*ωa*/2πc) for both polarizations, corresponding to 1.466 × 10^10^~1.468 × 10^10^ Hz in the microwave band. The bandwidth of the proposed PIC is not so wide compared to that of single-polarization circulators reported in literature^13,14^. However, some measures can be taken to increase the bandwidth. First, some methods, such as inserting air holes and dielectric cylinders in the waveguides and central cavity, and using MO materials in triangle shape or hollow cylinders in the cavity, can be adopted to achieve better impedance matching between the waveguides and the central cavity. As a result, the quality factor of the central cavity will be decreased and thus the bandwidth will be increased. Besides the influence of the cavity quality factor on the bandwidth, the balance between the responses of ferrite for TE circulation and plasma for TM circulation also has influence on it. For this, we can apply different external magnetic fields to the ferrite and plasma materials respectively for better balance and thus the common bandwidth of TE and TM waves can be increased. This method, however, requires more sophisticated technique to control the applied magnetic field. Furthermore, with the development of modern technology, new MO materials with more powerful rotation effect can be expected in the future and then the bandwidth of the circulator can be improved accordingly.

**Figure 9:**
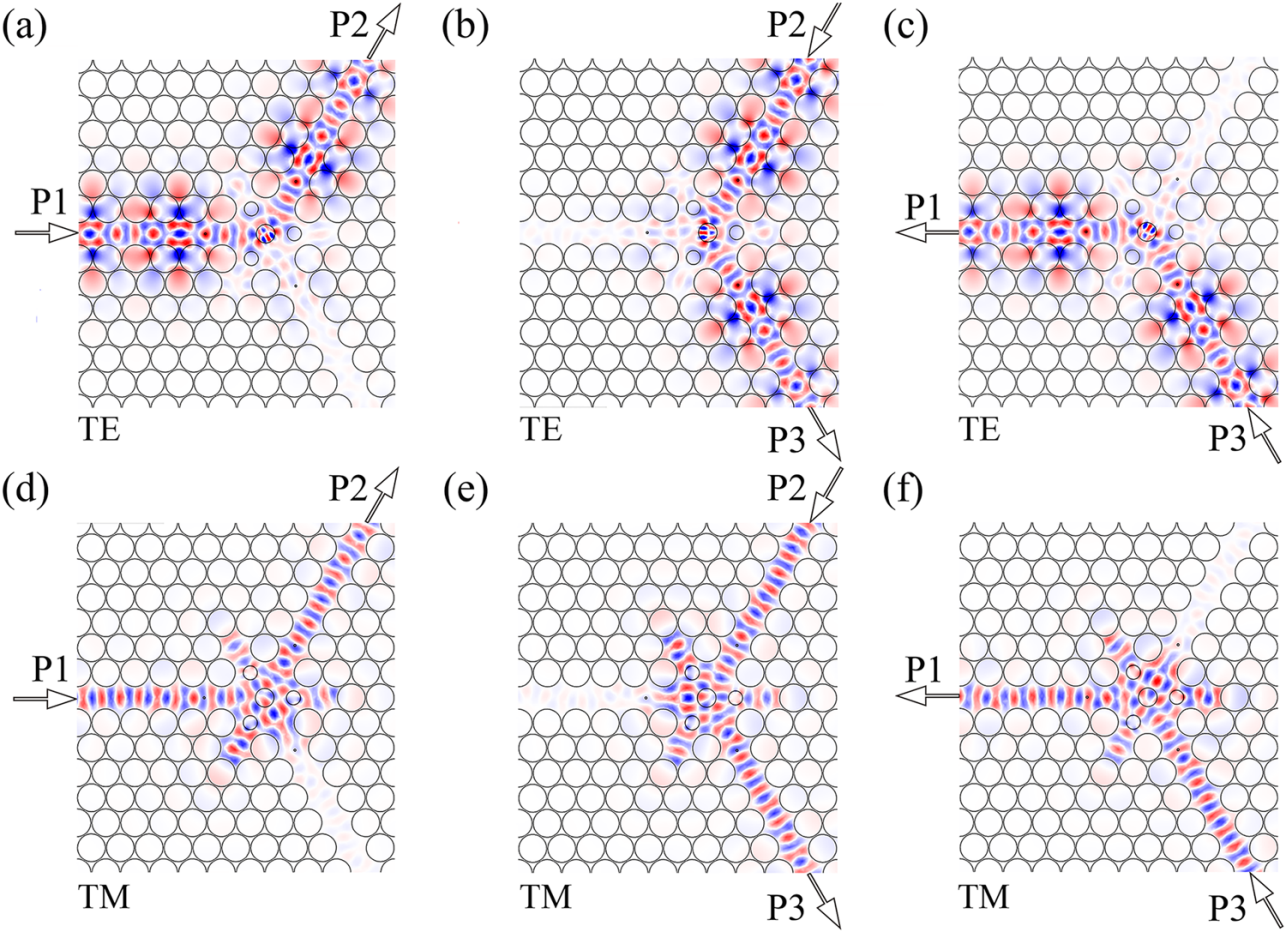
Distributions of electric field *E*_z_ in the centre region of PIC circulator for TE wave (**a**) input from P1, (**b**) input from P2, (**c**) input from P3; distributions of magnetic field *H*_z_ for TM wave (**d**) input from P1, (**e**) input from P2, (**f**) input from P3.

To verify the feasibility of the proposed PIC, the field distributions in the centre region of the PIC are plotted at the operating frequency of 0.48895 (*ωa*/2πc), corresponding to 1.467 × 10^10^ Hz, as shown in Fig. 9. Figure 9 shows that the structure does realize the expected function. For both polarizations, the input waves are launched from the input port and are transmitted almost completely to the output port, with almost no power being transmitted to the isolated port. The ferrite and plasma materials used provide rotation effects for the TE and TM polarizations, respectively. By comparing Fig. 9(a–c) with Fig. 4(b–d), it can be found that most of TE wave from the input port is rotated by the centre ferrite material, and outputs from the output port. Only a small part of the wave is distributed among the three resonant cavities. As a result, these three resonant cavities and the plasma materials have only a slight effect on the TE wave, in that the field distributions of the TE wave in the PIC are similar to that in the TE PhC circulator. Similarly, comparing Fig. 9(d–f) with Fig. 6(b–d), the field distributions of the TM wave in the PIC are similar to that in the TM PhC circulator. These results demonstrate that the proposed PIC works quite well for both polarizations.

## Conclusion

In conclusion, we have proposed and investigated a type of PIC based on plasma and ferrite materials in 2D PhC slab. By analyzing the wave equations in ferrite and plasma materials, we get the idea to build the TE PhC circulator with ferrite and the TM PhC circulator with plasma, respectively. Then, the PIC is designed by properly combining these two types of circulators together. For the TE-polarization circulator, the lowest insertion loss obtained is 0.05 dB and the isolation can reach 25.94 dB; for the TM-polarization circulator, the lowest insertion loss and corresponding isolation obtained are respectively 0.14 dB and 21.0 dB. For the PIC, the insertion loss and isolation obtained are slightly worse than that in the TE and TM PhC circulators, but they are respectively more than 20 dB and less than 0.15 dB for both polarizations. Such a structure is useful for improving the efficiency and reliability in polarization-independent devices for large-scale integrated photonic circuits.

## Method

Numerical simulations are performed by using finite-element method in the RF module included in COMSOL Multiphysics. The band structure in Fig. 3 is obtained by the study of Eigen-frequency in the triangle-lattice PhC. The transmission characteristics and field distributions in Figs 4, 6, 8 and 9 are obtained by the frequency-domain analyses of our model. All the parameters are optimized by the Nelder-Mead optimization method.
